# Stakeholder-specific adoption of AI in HRM: workers’ representatives’ perspective on concerns, requirements, and measures

**DOI:** 10.3389/frai.2025.1561322

**Published:** 2025-05-30

**Authors:** Christine Malin, Jürgen Fleiß, Stefan Thalmann

**Affiliations:** Business Analytics and Data Science-Center, University of Graz, Graz, Austria

**Keywords:** artificial intelligence, workers’ representatives, requirements, measures, HRM

## Abstract

**Introduction:**

AI regulations aim to balance AI’s potential and risks in general and human resource management (HRM) in particular. However, regulations are not finally defined and the perspectives of key stakeholders of HRM applications are not clear yet. Research on AI in HRM contributes only to a limited extent to the understanding of key HRM stakeholders, and the perspective of workers’ representatives is especially lacking so far.

**Methods:**

This paper presents a study of three focus group workshops investigating workers’ representatives’ perspectives, to determine which concerns they perceive when using AI in HRM, which resulting requirements they have for adopting AI in HRM, and which measures they perceive as most suitable to fulfill them.

**Results:**

Our results revealed that workers’ representatives were critical of using AI across all HRM phases, particularly in personnel selection. We identified requirements and measures for adopting AI in HRM from the perspective of workers’ representatives. These were summarized in a catalog including six dimensions: control, human oversight, responsibilities, transparency and explainability, lawful AI, and data security.

**Discussion:**

Our findings shed a nuanced light on workers’ representatives’ needs, providing relevant insights for research on stakeholder-oriented adoption of AI in HRM and for specifying current AI regulations.

## 1 Introduction

Artificial intelligence (AI) is increasingly being used for various tasks in human resource management (HRM), but at the same time the associated risks, such as biased decision-making, are intensely debated (Prikshat et al., 2022). To ensure responsible use of AI, legislators worldwide aim to address risks of AI use with regulatory efforts. The Artificial Intelligence Act (AI Act) of the European Commission is one of the most comprehensive AI regulations worldwide. The AI Act classifies HRM as high-risk area and establishes several specific requirements for using AI in HRM (European Parliament, 2024). After entering into force in August 2024, concrete implementations and requirements are currently being developed in standardization committees. An explicit goal of this process is to align the detailed specifications with relevant stakeholder expectations.

For AI applications in HRM, stakeholders can be distinguished in internal stakeholders who can directly influence HRM processes (e.g., HR professionals). Then there are external stakeholders with influence (e.g., regulators) or those who are affected by HRM decisions (e.g., applicants). Finally, there are stakeholders that represent the interests of the workforce (e.g., representative bodies such as workers’ representatives). Hence, when aiming to align specific design of the AI Act requirements concerning AI use in HRM with relevant stakeholders’ expectations, all three stakeholder groups have to be considered. All those stakeholders have an interest in shaping the use of AI in HRM processes, as the workforce is influenced by HRM processes. This includes, e.g., determining the qualification and diversity of the workforce through strategic personnel selection (Stone et al., 2015), by promoting skills, professional development, and loyalty to the organization through targeted onboarding and training measures (Cahyani et al., 2025; Mosquera and Soares, 2025) and by increasing job satisfaction through fair remuneration measures (Haiedar and Kholifah, 2025). Furthermore, HR practices have an impact on job satisfaction, organizational culture, employee retention, and turnover (Afzal et al., 2025; Wardhani and Yanti, 2025).

Research on adopting AI in HRM also increasingly uses a stakeholder-oriented approach, especially when developing solutions intended to foster successful adoption. Those solutions often provide an analytical concept of AI adoption frameworks, taking the form of broad, but at the same time general, overviews of the requirements of different stakeholder groups (Prikshat et al., 2022). However, this poses challenges such as proposing imprecise and conceptual recommendations for action, resistance from stakeholders and insufficient consideration of legal and ethical aspects. Furthermore, these challenges are intensified by previous empirical studies aimed to reconstruct the perspectives of individual stakeholder groups, focusing primarily on internal (e.g., Malin et al., 2023) or external key HRM stakeholders (e.g., Fleiß et al., 2024). The perspectives of representative bodies such as workers’ representatives as the third key HRM stakeholder group have so far been neglected in the literature on adopting AI in HRM. However, this stakeholder group is crucial for several reasons: (1) it is the link between employees and employers to ensure that AI is used in a way that meets the interests of both, and (2) it has a (legally) guaranteed say in the adoption of AI in the organization (e.g., in Austria, §59 Labour Constitution Act).

A stakeholder-oriented approach, as adopted in both the development of the AI Act and the scientific literature, offers a promising lens for investigating the requirements of key HRM stakeholders in general and of workers’ representatives, in particular (Freeman, 2010). We investigate organizational and technical requirements of workers’ representatives regarding the adoption of AI in HRM in three consecutive focus groups workshops with workers’ representatives. We also derive and evaluate promising measures to meet the requirements.

Our results contribute to understanding the perspective of workers’ representatives. We demonstrate to what extent and why they perceive using AI as critical for various HRM tasks and reconstruct factors influencing requirements for adopting AI. Our findings also advances research on AI in HRM by identifying and prioritizing the core requirements for adopting AI in HRM from a previously (empirically) unresearched key HRM stakeholder group, while also providing promising measures to fulfill them. We identified organizational and technical concerns when using AI in HRM, which vary in perceived criticality in HRM tasks. Based on these concerns, requirements, and measures for adopting AI were developed, which can be assigned to six dimensions: control, human oversight, responsibilities, transparency and explainability, lawful AI, and data security. Our results also provide relevant insights for legislators for the AI Act’ s specification.

## 2 Background

### 2.1 AI in HRM

The discrepancy between the high potential of AI and its risks is currently the subject of controversial debate both in academia (Pan and Froese, 2023) and in practice. AI’s ability to learn from given data sets (Huang and Rust, 2018) enables its use in various fields, including the detection of cyber threats (Qiquieh et al., 2025), or security management in mobile edge computing (Alzubi et al., 2023). Another promising application area is HRM, where AI can support or completely take over a wide range of HRM activities in all stages of the employee life cycle (ELC) (Budhwar et al., 2022). The ELC represents eight successive HRM phases that future and existing employees progress through when interacting with the (potential) employer (Gladka et al., 2022).

The first ELC-stage, *search and discover*, includes labour market analysis and planning of current and future job needs (analysis and development job profile). AI can support this, for example, by analyzing and neutralizing existing job advertisements for gender-specific differences (Laurim et al., 2021).

The second ELC-stage, *consider and apply*, describes the analysis and response to job postings and the search for candidates (job description). Here, for example, an AI-based job search system is able to select the most suitable candidate from a candidate pool for an advertised job position by comparing a job offer with (several) user profiles (Ochmann and Laumer, 2020).

The third ELC-stage, *assess*, includes conducting interviews and the selection of applicants (personnel selection), in which AI can derive job-relevant soft skills and personality traits from application data (e.g., CV) with the help of machine learning and/or natural language processing (Laurim et al., 2021).

The fourth ELC-stage, *accept*, includes integration into the company, contract preparation and signing (preparation for starting work). For example, AI can generate contracts based on user promotions (Williams, 2025).

The fifth ELC-stage, *explore*, describes introducing employees to the organizational culture, training them and setting up the work environment (onboarding). Here, AI can be used to check documents or conduct compliance training (Choudhary, 2022), as well as using chatbots to provide new employees with information about their day-to-day work (Kaushal et al., 2023).

The sixth ELC-stage, *build-up*, describes performance appraisals, acquiring and ensuring the required skills to perform core tasks (development and training). AI is able to support this stage by analyzing employee performance using predefined performance parameters or identifying trainer characteristics such as personality types and suggesting tailored training programs based on them (Maity, 2019).

The seventh ELC-stage, *maturity*, describes the long-term retention of employees and monitoring satisfaction and performance (retention and HR analyses). Potential AI areas of application in this ELC stage include predicting employee turnover (Zhao et al., 2018) or developing objective algorithms for salary determination, performance appraisal and salary adjustment using AI (Escolar-Jimenez et al., 2019).

The eight and last ELC-stage, *repeat or decline and leave*, describes the departure of employees from the company (separation and exit management).

While the application of AI supports various HRM activities, it entails various challenges. These range from opacity (Langer and König, 2023), small training data sets (Tambe et al., 2019), lack of security (Mohapatra et al., 2023), discriminatory decisions, and overreliance on AI-based decisions (Malin et al., 2024). Risks that can have serious consequences for various HRM stakeholders, resulting in a negative public perception of AI. Furthermore, these challenges can lead to conflicts and disagreements between various HRM stakeholders. For example, the opacity of AI prevents HRM stakeholders from understanding HRM decision-making processes, which reinforce their distrust of the technology (Park et al., 2021). If AI is trained with biased training data, resulting HRM decisions can lead to legal disputes with affected employees or applicants and create tensions between HR professionals and those responsible for inclusion and diversity (Harper and Millard, 2023). Furthermore, there is a risk that AI systems that have been trained with too small data sets reduce the reliability of AI and reduce trust in HRM decisions. The processing of personal data plays a major role in HRM, which is why security and data protection risks can promote compliance conflicts with data protection officers and supervisory authorities. In the short term, these challenges can lead to employee resistance to adopting AI, loss of trust and low employee satisfaction, and in the long term, they pose legal risks such as data protection breaches, discrimination claims, reputational damage and regulatory sanctions that affect both the company and its employees.

### 2.2 Regulation of AI in HRM

One promising approach to managing HRM-specific risks of AI are HRM-specific regulations (Alzubaidi et al., 2023), as currently pursued by legislators worldwide. Efforts include the AI Bill of Rights in the United States of America, the New Generation Artificial Intelligence Development Plan in China, the UK National AI Strategy in the United Kingdom, and the AI Act in Europe. Regulatory efforts around the globe focus on different areas in the field of HRM. In the United States of America, the AI Bill of Rights introduces guidelines to protect individual rights, while some states such as New York have specific but mandatory requirements for recruitment. In China, the focus is on efficiency and innovation in the working environment without imposing strict regulations, while the United Kingdom emphasizes voluntary ethical guidelines for the use of AI in HRM. In contrast, in the European Union, the AI Act calls for binding regulations on data protection and anti-discrimination in the context of HRM. Here, the AI Act follows a risk-based approach and categorizes HRM as high risk and therefore several specific requirements must be met by AI. A high-level Expert Group on AI (AI HLEG) developed ethical guidelines, directives, and policy and investment recommendations, and a rating list for responsible AI (European Commission, 2024b). The legislative steps in the field of AI based on the recommendations from the AI HLEG. (European Commission, 2024a). The AI HLEG’s advisory function in its original form has been completed following the adoption of the AI Act, however, a stakeholder approach is still being pursued. A stakeholder approach is characterized by considering the interests and needs of all relevant stakeholders and including them in the (strategic) decision-making process, and striking a balance between their requirements, leading to a positive response and long-term success (Freeman, 2010). The AI Act’s requirements are being specified by the European Commission, regulatory authorities, and standardization committees. To specify the HRM-specific AI Act regulations, the concerns and requirements of key HRM stakeholders when adopting AI in HRM must be understood and addressed.

### 2.3 Research on AI in HRM

In the HRM literature, key stakeholders are categorized and examined in varying detail. HRM models, such as ELC (Gladka et al., 2022), provide a more high-level view, while context-dependent studies often analyze key HRM stakeholders in more detail (e.g., Schuler and Jackson, 2014; Langer and König, 2023). Generally, three types of key HRM stakeholders tend to emerge in these studies: (1) internal stakeholders such as employees, who are directly active in the company and influence HRM strategies, (2) external stakeholders such as applicants, who do not play an active role in companies, but influence HRM decisions, and (3) representative bodies such as workers’ representatives.

Since a stakeholder approach helps to understand and manage the impact of AI on the various key HRM stakeholders, it has gained relevance in understanding the adoption of AI in HRM (Prikshat et al., 2022). The literature increasingly offers stakeholder-centered approaches (Park et al., 2022) and frameworks that aim to promote the adoption of AI in HRM. These are particularly relevant for the specification process of the AI Act regulations and thus for adopting AI, as they contribute to the understanding of the stakeholder groups. However, these are mainly characterized by a conceptual approach and deal with the requirements of HRM stakeholders at a high-level (e.g., Prikshat et al., 2022) or focus mainly on internal stakeholders such as employees (e.g., Park et al., 2022). This unbalanced distribution of attention is also reflected in the general research on investigating the perspective of HRM stakeholders of AI, which primarily focuses on the requirements of internal (e.g., Malin et al., 2023) and external stakeholders (e.g., Fleiß et al., 2024). The perspective of the third key HRM stakeholder group, representative bodies, has been neglected in general, but especially regarding workers’ representatives, both in research on conventional HRM (Bondarouk and Brewster, 2016) and AI-based HRM. One possible explanation for the missing perspective of workers’ representatives is that it is challenging to gain access to this particular stakeholder group. This lack of attention is problematic for the AI Act’s specification endeavour and the general adoption of AI in HRM. Thus, the successful adoption of AI in HRM and the implementation of the AI Act requires an understanding of all relevant stakeholder requirements, including those of workers’ representatives. We extend previous conceptual research on HRM-specific AI adoption frameworks by focusing on a specific and previously neglected key HRM stakeholder group and empirically investigating their requirements for adopting AI. We take a more nuanced look at the concerns and requirements of workers’ representatives and propose measures to fulfill them.

## 3 Materials and methods

**Figure 1:**
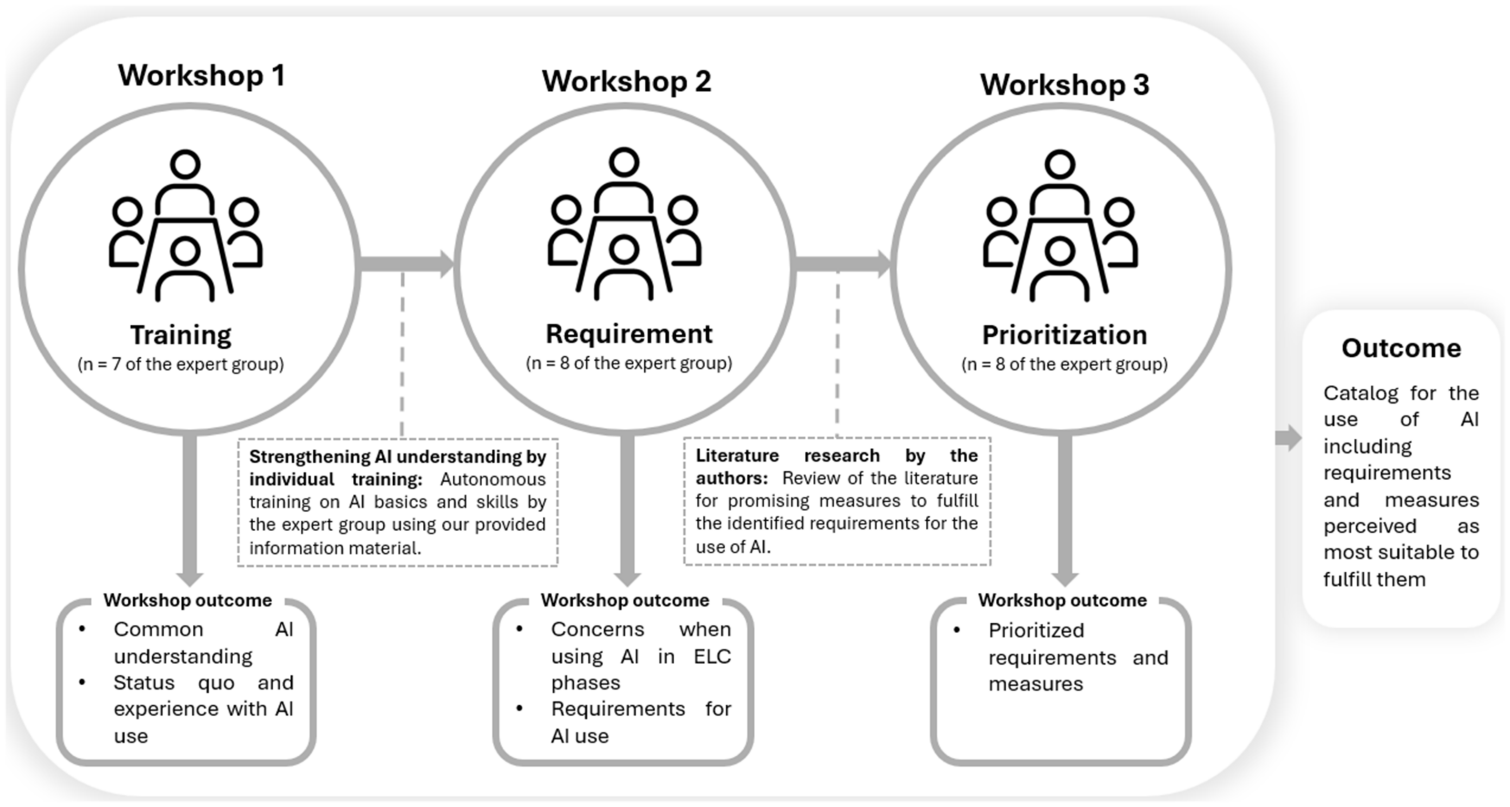
Overview of the research process.

Our study aims to identify core requirements and the most promising measures for adopting AI in HRM from the perspective of workers’ representatives through a stakeholder-oriented approach. Focus group workshops are a promising instrument in applying a stakeholder-oriented approach, as they enable the identification of stakeholder needs (Huemann et al., 2016). Furthermore, due to the interactive exchange among the workshop participants, focus group workshops are better suited to depict collective opinion and consensus-building processes than other qualitative research methods such as interviews, and they also provide deeper, more contextualized insights into their specific concerns and requirements than quantitative methods such as surveys (Longhurst, 2016; Nyumba et al., 2018; Queirós et al., 2017). Therefore, we conducted three focus group workshops with an expert group of 12 prospective and active workers’ representatives between December 2023 and June 2024 (see Figure 1).

Expert selection for the focus group workshops was conducted with a representative of an umbrella organization of trade unions also helping in selecting the experts to ensure a broad range regarding company size, industry sector, and level of experience. The experts came from diverse sectors, ranging from education to steel processing. Most of the experts already had several years of experience as workers’ representatives and had the role of chair at the time of the study. All five experts not actively involved in the works council at the time of the study were already in training at trade union schools. Table 1 gives an overview of the experts.

**Table 1:** Demographic details of the expert group.

ID	Industry	Workers’ representatives function
E1	Semiconductor manufacturing & education and research	Member and cashier
E2	Steel processing	In training
E3	Warehouse logistics and automation	Member
E4	Public employment services and labour market policy	In training
E5	Employee representatives and interest groups	In training
E6	Social economy	Chairman
E7	Public employment services and labour market policy	Member & in training
E8	Personnel services	Vice-chairman
E9	Trade unions and employee representatives	Chairman
E10	Education	Chairman
E11	Trade unions and employee representatives	No member
E12	Pulp and paper	Chairman

While the first and second focus group workshops were conducted in a face-to-face setting, the third was conducted online, all lasting approximately 120 min each. All focus group workshops were recorded and transcribed using aTrain (Haberl et al., 2024). Quotes used in the article were translated from German to English.

The first focus group workshop with the expert group (*n* = 7), a *training workshop*, took place in December 2023. The aim was to ensure that all experts had a common base of knowledge and understanding of AI. First, we discussed the expert’s understanding of AI and the status quo of AI use in HRM leading to a common consensus on the basics and capabilities of AI within the expert group. After the focus workshop, we provided the participants with relevant information material on the basics and features of AI. A period of 3 months was planned between the focus group workshops so that the expert group had sufficient time to (re)work through the information material independently. The main outcome of the training workshop was to ensure a common understanding of AI within the expert group.

The second focus group workshop (*requirement workshop*) with the experts (*n* = 8) took place in March 2024. This workshop aimed to identify the requirements of workers’ representatives so that they would adopt AI in HRM and to understand their underlying concerns. The requirement workshop consisted of brainstorming, clustering, voting, and in-depth discussion regarding (1) the perceived criticality of AI use in each ELC stage and (2) requirements for adopting AI in HRM.

The workshops’ transcript were analyzed using the variant of thematic analysis from Braun and Clarke (2006). We identified patterns in the data and then categorized the data extracts into groups, resulting in initial codes. For example, the data revealed skepticism regarding the comprehensibility of AI-based decisions and unintentional discrimination, particularly in personnel selection, strengthening the need for transparent and explainable AI systems. Therefore, “transparency and explainability” became a code. Related codes were grouped into topics that were refined, continuously expanded, named, and verified in an iterative process. For example, the codes “transparency and explainability” and “data security” were assigned to the topic “technical requirements.” The central outcome of the focus group workshop was the identification of the perceived criticality of AI in HRM, and technical and organizational requirements for adopting AI.

A period of 3 months (March to June 2024) elapsed between the second and third focus group workshops, during which we conducted a literature search to create an overview of state-of-the-art measures already proposed in the literature that addressed the identified requirements (second workshop). We reviewed the literature for measures that fulfill the aspects of the requirements identified. As the identified measures from the literature addressed the identified requirements to varying degrees, we divided the identified requirements into three levels of requirements fulfillment: (1) *low level*, (2) *medium level*, and (3) *high level*. We assigned the measures identified in the literature to the respective levels.

To discuss and prioritize the identified requirements (see requirement workshop) and measures (see literature research), a third focus group workshop (*prioritization workshop*) was conducted with the experts (*n* = 8) in June 2024. The prioritization workshop aimed to determine whether and which measures provided in the literature are accepted by the expert group or considered most suitable to meet their requirements for adopting AI in HRM. We discussed the organizational and technical requirements individually, which were identified during the second focus group workshop. We asked the expert group which of the organizational or technical requirements identified in the second focus group workshop were most important to them, and we asked them to prioritize them by again assigning points (the more points, the higher the importance).

As in the second workshop, the transcripts from the third focus group workshop were analyzed using thematic analysis (Braun and Clarke, 2006). The same coding and analysis scheme was used. The key outcome of this workshop was that the expert group identified and prioritized the requirements for adopting AI and the measures that were perceived to be most suitable to fulfill them, which we summarized in a catalog.

## 4 Results

In our focus group workshops, the use of AI in HRM is generally perceived critically, with concerns varying for the different ELC stages and being expressed to varying degrees. These concerns are particularly pronounced in ELC stages focused on existing employees. This indicates a possible influence of the target group orientation of workers’ representatives (existing vs. future employees) of the stages on the perceived criticality.

*Personnel selection* was named as the most critical area of AI application, as it is assumed that AI’s self-learning ability leads to a lack of objectivity and (un)conscious discrimination. The self-learning ability of AI refers to the ability of currently existing AI models to improve their performance by fine-tuning with curated data within a predefined scope (i.e., artificial narrow intelligence). Furthermore, the neglect of human oversight, the inability of AI to replace human skills, and a lack of transparency and limited accountability were also raised as concerns. The ELC stages *retention and HR analytics* and *development and training* were named as the second and third most critical areas of application respectively, with both due to concerns about the perceived lack of measurement options, the loss of personal expertise and control, and the perceived technology readiness. The ELC stages *job description and separation* and *exit management* were both categorized together as fourth-critical areas of application. The former can be attributed to the perceived low technology readiness, while the latter is due to the perceived risk of AI sending attractive job offers for (competing) companies to current employees. The other ELC stages were not named by any of the experts, indicating that the use of AI in these stages is perceived as uncritical.

Considering the stakeholder-oriented approach of the AI Act, the varying concerns mentioned reflect the specific interests of workers’ representatives, resulting in their requirements for adopting AI in HRM. We analyze to what extent and why workers’ representatives have concerns when using AI. We present the resulting requirements for adopting AI in HRM. We also identified promising measures in the literature for each requirement that fulfills it to varying levels. Specifically, we divided each requirement into three levels of fulfillment: (1) *low level*, (2) *medium level*, and (3) *high level*. Then we assigned corresponding measures to each level. The individual levels build on each other, meaning that each subsequent level includes the measures of the previous level(s).

**Figure 2:**
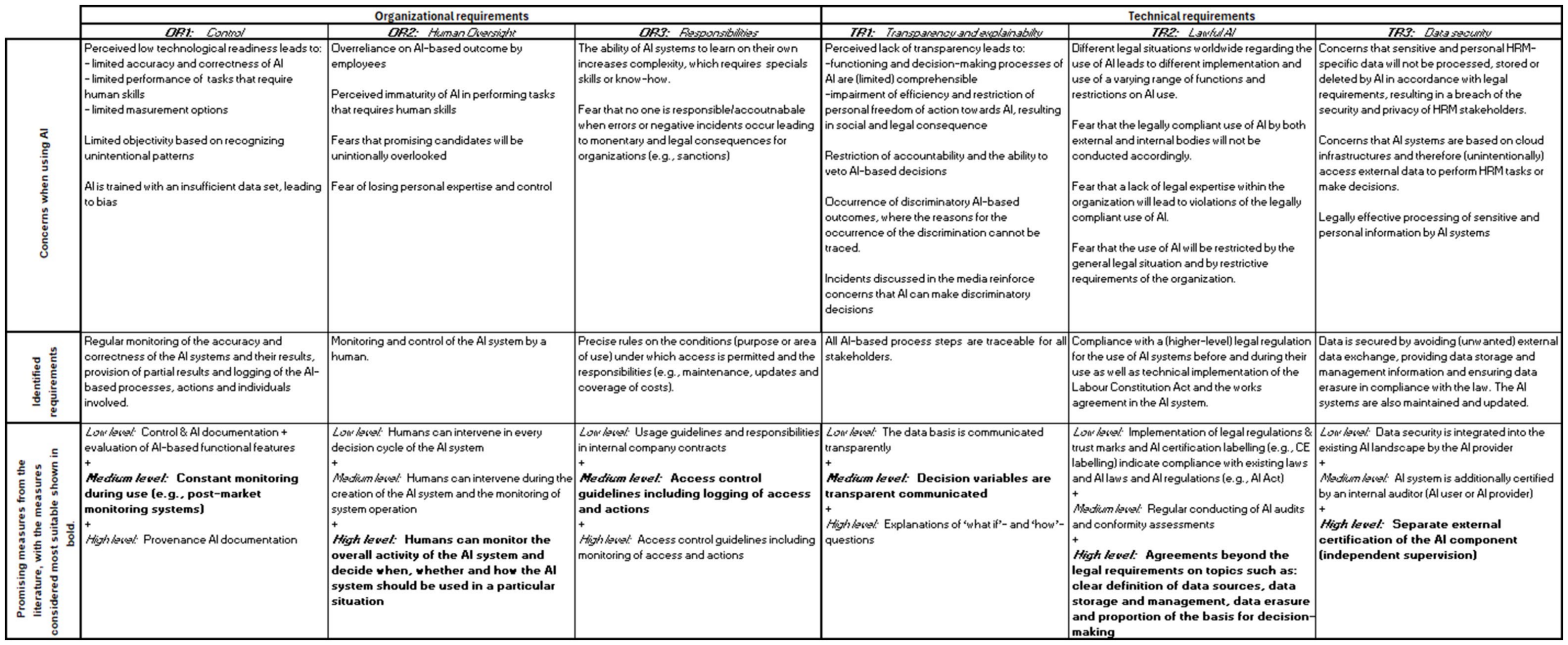
Stakeholder-oriented catalog containing concerns when using AI in HRM as well as core requirements and promising measures for the adoption of AI in HRM.

Summing up, we introduce a stakeholder-tailored catalog for adopting AI in HRM, including requirements and promising measures to fulfill them. We identified six core stakeholder requirements for adopting AI from the perspective of workers’ representatives, three of which can be divided into organizational (OR1 to OR3) and technical (TR1 to TR3). An overview of the concerns when using AI in HRM, the resulting requirements, and promising measures from the literature is shown in Figure 2. It also highlights measures that workers’ representatives consider most suitable for fulfilling the requirements.

### 4.1 Organizational requirements and measures

We identified three organizational requirements (OR1 to OR3) for adopting AI from the perspective of workers’ representatives: (1) responsibilities, (2) control, and (3) human oversight. Each requirement is explained in a separate sub-section, all following the same structure: First, we describe the concerns raised by the workers’ representatives in the second focus group workshop regarding the use of AI in HRM, highlighting those ELC stages in which these are most pronounced. Second, the resulting requirements for adopting AI in HRM, which we identified in the third focus group workshop, are presented. Finally, we introduce measures from the literature that fulfill these requirements. Building on the results of the third focus group workshop, we explain which of these and why the workers’ representatives perceive them to be the most suitable for fulfilling them.

#### 4.1.1 OR1: control

In our focus group workshops, even though the potential of AI for HRM is generally perceived to be high, it is also associated with limited technology readiness, which limits the accuracy and correctness of AI. Due to this perceived barrier, various consequences are feared by the expert group in almost every ELC stage. These concerns are particularly pronounced in the ELC stages *personnel selection*, *retention and HR analyses*, and *development and training*.

For *personnel selection*, concerns about the perceived low technology readiness of AI relate to two key implications: (1) the objectivity of the technology is limited, and (2) tasks that require human skills like moral judgment, empathy, and intuitive understanding which cannot be performed by AI. It is assumed that these tasks are based on conscious experiences and emotions that cannot be replicated by data. There are fears that AI’s ability to learn within a specific context could lead to (unintentional) patterns being recognized in the data, which could limit the objectivity of AI:

*When it comes to personnel selection, you’re bound to make discriminatory mistakes. Because you can’t train the AI to remain objective. [E10]*

The perceived limited objectivity of AI is viewed very critically, especially in *personnel selection*, as the expert group feared that it would lead to inaccurate results, which may result in suitable candidates being unintentionally rejected or not considered. For the organization, the potential loss of the most suitable candidates can mean lower efficiency and costs in the form of necessary retraining. Even if there are concerns about limited objectivity due to AI, it is assumed that this is higher in direct comparison to humans:

*I don’t entirely agree that it is more critical when a human makes a selection than when a computer makes one, because I actually believe that if it were possible, a computer would probably be more objective. [E7]*

This quote indicates that despite the perceived barriers of AI, its potential is still seen in its use. It also indicates the high level of trust in the accuracy and correctness of AI and skepticism towards human subjectivity.

Within *personnel selection*, concerns about the use of AI also relate to the perceived technology readiness to perform tasks that require human skills. In the focus group workshops, it was believed that AI is unable to recognize factors such as creativity, hold conversations, or perform tasks that require personal perception, leading to fears that promising candidates will be unintentionally overlooked:

*What I would wish is that an AI had looked over it objectively. I think these unique characteristics would probably often fall through the grid. It depends on the patterns […]. [E3]*

The perceived technology readiness is attributed to the fact that AI systems are trained with an insufficient data set, which does not provide it with enough information to recognize reliable patterns. It can lead to the learning of random biases as representative correlations:

*I mainly differentiate between personnel selection and development, simply because there is a risk of consciously or unconsciously failing to train AI correctly. […] but there are so many small things like recognizing dogs, wolves, what is a dog, what is a wolf, if the basic data is too small, there are enough examples, especially with image recognition. A wolf has always had snow in the background, so if the dog is in the snow, it’s a wolf. [E1]*

This expert highlights the risk that AI can be trained incorrectly, leading to erroneous outcomes. Thus, it highlights the fear that an AI will recognize distorted patterns due to an inadequate database and produce inaccurate decisions. Besides *personnel selection*, concerns of the expert group about the perceived technology readiness are also pronounced in other ELC stages such as *retention and HR analyses* and *development and training*. The perceived high criticality of using AI in *retention and HR analyses* lies in perceived limitations regarding measurement options. It is feared that there is a risk that if evaluation criteria are not clearly defined in advance, AI will perform the analyses with randomly chosen variables. It is also assumed that although AI can perform number-based measurements and analyses, it cannot include individual factors tailored to employees. Again, the concerns indicate a perceived low technology readiness of taking over human skills.

Similarly, the use of AI in *development and training* is associated with skepticism towards measurement options. In the focus group workshops, it was assumed that companies will benefit from using AI, as overall performance can be increased with the help of AI, however, there are also concerns that in the context of *training and development*, AI is only able to respond to individual personalities/developments to a limited extent. The above-mentioned concerns mean that control is named by the expert group as the most important organizational requirement. This requirement describes the perceived need to monitor AI systems continuously and their results regarding accuracy and correctness. The extent of this monitoring includes two elements: (1) It is assumed that HRM-specific AI systems can be evaluated, for example, by using fictitious reference persons to check the quality of decisions and that access to partial results is available. (2) The course of the AI-based processes, the actions conducted, and the people involved should also be logged:

*There is a protocol for the whole thing or process. We have something similar with a security tool, in that we know at any time how many people or who is in the company and who is in what area. […] But as soon as someone requests the log, I get an e-mail that the log has now been requested. Normally there are only the security forces, task forces, the fire brigade, and so on, but there, I can no longer check where I call the place where it is requested, who is the person who has requested it. [E12]*

This expert highlights that he or she would like to have a protocol for the whole process so that employees’ activities can be monitored. It highlights the complexity of the requirement as well as the strong desire of the expert group to be able to influence and monitor AI. One of the main reasons for the perceived need lies in the skepticism of the expert group that even if internal company agreements have been made, for example in the form of (supplementary) company agreements, these will not be implemented without the existence of external institutionalized control bodies. The expert group highlighted that ongoing monitoring takes place during use throughout the entire process and not just before the decision is made.

We have reviewed the literature to identify possible alleviating measures for the described concerns. We identified three measures that fulfill the identified control requirement from the workshops to varying degrees: To fulfill the control requirement at *low level*, the use of AI documentation in combination with evaluating AI-based functional features is promising. AI documentation records development processes, algorithms, data sets, test cases, and evaluation metrics while AI models are designed and validated (Tao et al., 2019). This provides a detailed description of the design and implementation of the AI models used (model architecture) and information about the training data, including the sources and pre-processing steps. Furthermore, the test methods used to evaluate AI in terms of performance and robustness are also documented. These factors lead to increased trust in AI (Bedué and Fritzsche, 2022). A further measure that goes beyond AI documentation, including the evaluation of AI-based functional features (*low level*), involves ongoing monitoring of the AI systems during use (*medium level*). This can be implemented with the use of post-market monitoring systems that document and analyze the performance of the AI systems throughout their entire life cycle (Mökander et al., 2022). This approach increases conformity and transparency, which is an essential element of trust in AI (Bedué and Fritzsche, 2022). *High level* is characterized by provenance AI documentation. Provenance AI documentation documents the course of an AI process step by step, showing who did what, when, and where. This documentation enables transparency, traces the origin of the data, shows the steps of data processing, and evaluates the trustworthiness of the results.

We discussed with the expert group the extent to which the measures identified in the literature are perceived as suitable for fulfilling the control requirement. There was a consensus within the expert group that the ongoing monitoring (*medium level*) was considered the most suitable measure to fulfill this requirement. This perception was justified by the expert group assuming that additional services (documentation) would not be utilized despite their benefits:

*I am also satisfied with the ongoing monitoring during operation. […] documenting this entire provenance and always doing so is likely, but again, nobody would look at it, it would be more of a theoretical construct. [E3]*

This expert describes the documentation of the entire provenance as a theoretical construct and indicates that it would not be used by the organization. Even though a consensus emerged within the expert group regarding the suitability of *medium level* for fulfilling the control requirement, *high level* and *low level* were also discussed as alternative suitable measures. Within this discussion, it was emphasized that AI can only be controlled if the origin of the data is traceable and visible (*high level*).

#### 4.1.2 OR2: human oversight

The expert group discussed the use of AI in HRM in the context of neglecting the human factor. It usually includes three perspectives: (1) loss of human oversight, (2) need for human involvement, and (3) human rights perspective. Although these discussions occur across all ELC stages, they are most pronounced in *personnel selection*. This discussion is based on concerns about failing to ensure that AI-based (personnel selection) decisions are scrutinized by human decision-makers:

*You can’t train the AI to remain objective. And as an organization, you’re probably missing out by having a human overlook it. [E10]*

This expert highlights the risk that organizations miss ensuring human oversight. This statement refers to two factors: First, it indicates that it is assumed that organizations may fail to monitor and ensure human oversight as a result of cost pressures or resource-based efforts. Second, the statement reflects the skepticism of the expert group that human HR professionals will question the AI-based recommendations and thus rely too much on the AI-based recommendations. While these fears relate more to conscious actions by humans, the other concerns mentioned relate to the perceived low technology readiness in performing human skills. It is believed that AI relies on hard facts (e.g., years of professional experience) when making decisions and is unable to recognize human components, leading to fears that promising candidates will be unintentionally overlooked:

*It’s simply the human aspect that falls short, the humanity. And everyone has actually said that these personal components simply drop out. If I now assume that I prove myself for a position and I am rejected because I only have two years of professional experience and don’t even get to the interview. But as a person, I would fit in well with the team. The head of assessment would hire me because I also have other skills that are necessary for this position. In my opinion, AI can’t recognize that. [E9]*

This expert highlights that it is assumed that, unlike humans, AI cannot recognize human components in applicants. This quote indicates that some experts believe that certain tasks still need to be carried out by humans. These tasks include, for example, the composition of internal organizational teams, as it is assumed that this requires human experience in considering common problems that arise when certain groups of people or cultures work together.

Concerns of the expert group regarding the neglect of human oversight and the human factor are not limited to the specific case of *personnel selection* but are also evident in the ELC stages *retention and HR analyses* and *development and training*. The fear of losing personal expertise and the ability to influence and reflect are particularly evident here:

*I think it’s also difficult with HR analyses if they are entirely AI-based, because otherwise there are usually a few employees who might make decisions together as a team. And it’s also important that the AI runs over it and that’s pretty much it. Because then you can make other decisions, because as a human also get new input and can say, okay, I didn’t see it that way, but that’s an interesting idea, let’s talk about it again. And that might get lost. [E5]*

This expert highlights that in conventional HRM processes, employees make decisions together as a team and thus (continuously) receive and discuss new input for the decision, whereas AI simply makes the decision based on the input it receives at the time of decision-making. This quote highlights the skepticism towards the fear of losing expertise and control. Based on these concerns, experts named ensuring human oversight as the second most important organizational requirement for adopting AI in HRM. Human oversight refers to the monitoring and controlling of AI systems by humans, which ensures accurate and correct AI systems:

*[…] which should be an organizational requirement - human sample analysis for negative decisions. In other words, perhaps a committee should make the decision. And accordingly, according to searches, learning questions, according to functions. [E1]*

This expert highlights that HRM decisions should be made by (human) committees. It indicates the expert group’s desire that AI should not neglect the human factor, whereby the final monitoring and decision-making should always be carried out by humans. From the expert group’s perspective, human oversight is not limited to human monitoring of AI. It should include two factors: (1) the ability to intervene in the AI-based process anywhere and at any time and (2) the human control function. They make it clear that the expert group closely associates human oversight with a sense of control. By being able to intervene in the AI-based process at any time, they counteract the feeling of a possible loss of control over AI and the perceived risk of AI. The desire for control is particularly pronounced in AI-based decisions in the final stage:

*Human oversight was the most important for me. The final oversight, so to speak, by humans in the whole picture. For me, it’s about a final control by humans in the whole picture […] but in the end, I still put the control by humans not to let anything slip away. [E8]*

This expert highlights the importance of the final decision being conducted by humans and that humans do not let anything slip through their fingers. It indicates the desire that the final decision should still be made by a human.

To fulfill the requirements of the expert group regarding the safeguarding of human oversight in HRM, we again reviewed the literature for measures. We found different types of human oversight are distinguished (Enqvist, 2023) and identified three measures that fulfill the human oversight requirement to varying degrees: *Low level* implies that the human can intervene in every decision cycle of the AI system (human-in-the-loop). Consequently, humans control and authorize every AI-based decision (Díaz-Rodríguez et al., 2023). At *medium level*, humans can also intervene during the development of the AI system and the monitoring of system operation (human-on-the-loop) (Díaz-Rodríguez et al., 2023). At *high level* the human can monitor the overall activity of the AI system and decide when, whether, and how the AI system should be used in a particular situation (human-in-command) (Díaz-Rodríguez et al., 2023).

To identify which of the measures proposed in the literature are considered by the experts to be the most suitable for the fulfillment of the human oversight requirements, we discussed these with the expert group. There was a consensus that the *highest level* (human-in-command) was considered most suitable for it. The expert group expressed the opinion that the AI system can only be controlled if humans can intervene at every step:

*Yes, who else, of course, human-in-command, from my perspective, the human should always have the possibility to overrule this in every step. [E3]*

The expert’s statement highlights the assumed importance of humans overruling AI at every step. Despite the consensus regarding *high level*, *low level* (human-in-the-loop) and *medium level* (human-on-the-loop) were also discussed as suitable measures. The reasons for considering these two levels were contradictory, as it was felt that it is sufficient if the human control function is only provided in the decision-making phase, while human intervention during the development phase means that potential risks can be adequately addressed in advance.

#### 4.1.3 OR3: responsibilities

In the focus group workshops, the ability of AI systems to learn on their own was often associated with increasing complexity across all ELC stages, which requires the deployment of trained professionals to maintain AI:

*When it comes to training development, I think a little care is needed, because I need a person, a competent person, to maintain the systems. [E7]*

The expert’s statement emphasizes the need for a competent person responsible for maintaining the AI. It indicates that the expert group assumed that the maintenance of AI systems differs from conventional HRM tools due to their complex mechanisms and peculiarities and requires special skills or know-how. These activities include, for example, the maintenance of AI systems or the implementation of updates. It is assumed that the appropriate execution of the activities can only be ensured by assigning specific responsibilities. However, it still seems unclear which persons are actually responsible for these activities and how high the corresponding costs are:

*I think because of AI, because of the responsibility - is there a separate staff? Is the IT department responsible? Is the HR department responsible? Or are both responsible? Who has access to the user system? And then I also have people in charge. What happens when wrong decisions or data leaks occur? Who has the final responsibility? [E5]*

The expert emphasizes that the allocation of responsibility to individuals or organizational departments is an open question, including who has access to the systems and who is specifically held accountable for wrong decisions or data leaks. This quote indicates that the clear regulation of responsibilities should not only ensure responsibilities, but also accountability. The latter is intended to ensure that there is no misuse and that a natural person can be held accountable when errors or negative incidents occur. This is because noncompliant use of AI will be associated with monetary and legal consequences for organizations, ranging from sanctions to employee dismissals.

Given the concerns regarding the unclear responsibilities and various consequences of AI, there is a growing demand by the expert group to know who has access to the AI system, when, and to which specific aspects (e.g., AI-based outcome). Similar to the responsibilities, the access authorizations also appear to be unclear but relevant.

Based on the above-mentioned concerns, responsibilities was named by the expert group as the third most important organizational requirement for adopting AI in HRM. Responsibilities is defined as defining precise rules on the conditions (purpose, area of use, etc.) under which access is permitted and the responsibilities (e.g., maintenance, updates, and coverage of costs). This requirement is based on concerns of the expert group about unclear responsibilities, accountability, access, and accountability. To address these concerns, we have identified three measures in the literature that fulfill the requirement to varying degrees: *Low level* includes the definition of access and usage guidelines (Du, 2024) in internal contracts such as role-level accountability contracts (Lu et al., 2024). Another measure, alongside the definition of these guidelines, involves logging access and controls (*medium level*). High level contains an active measure, as monitoring of access and actions is provided alongside the definition of guidelines and logging (Mylrea and Robinson, 2023).

The suitability of the measures was discussed with the experts regarding the responsibilities requirement. The expert group had a consensus that the definition of access including the logging of the action (*medium level*) is the most suitable for fulfilling this requirement. It was justified by logging access and actions to ensure time-delayed monitoring, as all necessary information (e.g., access by persons or actions performed) is thus available. The logging of additional information was considered superfluous, as it is assumed that it will not be accessed despite being available.

*I am of the opinion that if everything is actively monitored, then you need it. If it’s all logged, it is good to have, but if there is really active monitoring, there is just so much unnecessary data being produced that nobody looks at it. [E4]*

Although there was a consensus within the expert group in favor of *medium level*, the other two levels were also discussed as suitable measures. Their consideration was based on negative personal (professional) experiences in this context.

### 4.2 Technical requirements and measures

We identified three technical requirements (TR1 to TR3) for adopting AI in HRM from the perspective of workers’ representatives: (1) transparency and explainability, (2) lawful AI, and (3) data security. Similar to the organizational requirements, all technical requirements are presented in detail in individual sub-sections following the same structure.

#### 4.2.1 TR1: transparency and explainability

In the focus group workshops, AI systems are described as non-transparent across all ELC stages, meaning that their functioning and decision-making processes are barely or not at all comprehensible to humans. This characteristic of AI was viewed critically by the expert group, particularly in personnel selection decisions, as it makes it almost impossible for workers’ representatives to challenge the decisions made. AI can be reduced with a limitation of accountability and the possibility of using the veto against decisions:

*Afterwards you can’t understand how the decision was being made. […] So you can’t challenge it anymore. As a workers’ representatives I have no chance. I can’t challenge it anymore. [E1]*

This quote refers to the expert group’s fear that the lack of transparency of the AI system not only impairs its efficiency but also restricts their personal room for maneuver concerning AI, which is associated with social and legal consequences. This is because transparent and explainable AI-based systems were often discussed related to biases and discrimination. Incidents widely discussed in the media, such as the Amazon scandal when certain groups of people were disadvantaged in their AI-based personnel selection process, reinforce concerns of the expert group that AI can make discriminatory (personnel selection) decisions. This is due to the assumption that AI’s ability to self-learn can lead to a lack of objectivity and thus to (un)conscious discrimination (see OR1: Control—subsection). The expert group fears that biases slow down the objectivity of AI. The lack of transparency leads to the fear that AI-based decisions can not only be discriminatory but also that it is not possible to understand how discrimination arose in the first place. This, in combination with the perceived lack of transparency and explainability of AI systems, leads to a strong desire for gender-tested and nation-neutral AI systems, which is ensured, e.g., in the form of certifications.

Based on these concerns, the expert group named transparency and explainability as the most important technical requirement for adopting AI. This requirement is defined as the traceability of all AI-based process steps (data input, data processing, or outcome) for all HRM stakeholders. There is a need to ensure that the traceability of AI-supported processes during the ongoing operation of the AI system (AI-based decisions and the basis for decisions can be traced) and is also available during the training and development phase (methods and data sets used). Traceability is understood to mean that the respective HRM stakeholder groups, such as HR professionals or applicants, are provided with information in a way that they can understand about the task for which AI is being used, what the AI system does in each (decision-making) step and which (input) data is used for processing and decision-making. It should be possible to understand the underlying decision-making process, as described using the specific example of an applicant:

*[…] when I start the application process, as an applicant, it must be clearly communicated to me what is done with it, where it is stored. And exactly what happens with every process document that is created. This will probably be possible if I specify somewhere in a law that companies have to be forced to do this, otherwise they won’t do it. [E10]*

This statement indicates that the lack of transparency of AI systems is not only perceived by the expert group as a technical but also as an organizational challenge, whose solution requires implementing legal measures.

To address these concerns, we reviewed the literature for promising measures. The literature offers several measures to fulfill the requirement for transparency and explainability. We identified three measures that fulfill the requirements at three levels: *Low level* involves transparent communication of the data basis. Consequently, information about the input and output of the AI system is provided. Thus, explanations are provided about what an AI system has done or is doing and about the decisions made by the system (Sanneman and Shah, 2022). *Medium level* is characterized by transparent communication of the decision variables. Consequently, explanations are provided as to why an AI system has acted in a certain way or made a decision (Sanneman and Shah, 2022). *High level* provides counterfactual explanations, which means that explanations of ‘what-if’—and ‘how’—questions are available. Consequently, with the use of explainable AI (XAI) techniques, explanations are provided as to what an AI system would do under different circumstances or in a different context (Sanneman and Shah, 2022).

Aiming to identify the most suitable measure to fulfill this technical requirement, we discussed the measures identified in the literature with the expert group. A consensus within the expert group was visible that the transparent communication of the decision variables (*medium level*) was perceived as the most suitable measure that fulfills the requirement. This decision was justified by stating that by providing the decision variables, all necessary information is available to draw further conclusions for the AI-based decision or the process. In the focus group workshops, it was also highlighted that individuals interacting with AI have different technical backgrounds and understandings, which impacts their requirements for transparency and explainability of AI systems. While AI developers require detailed technical information for traceability, applicants tend to expect comprehensible explanations of the AI’s decision-making processes. At *medium level*, the expert group assumed that even laypersons are provided with sufficient information to understand the AI-based process.

Despite the consensus within the expert group regarding *medium level*, the higher level, which includes explanations for “what-if”—and “how”—questions (*high level*), was also discussed as a suitable measure. No one of the expert group considered transparent communication of the database (*low level*) as a suitable measure. This consideration indicates that the opacity of AI systems is perceived as a major technical challenge, which is why more comprehensive rather than limited disclosure of information about the AI system is desired.

#### 4.2.2 TR2: lawful AI

In the focus group workshops, the use of AI in HRM was associated with various legal concerns that arise across all ELC stages. Concerns relate to the different legal situations worldwide, which are clearly evident even within Europe. The different legal provisions in the individual nations mean that HRM tools are implemented differently and a varying scope of functions is used depending on the country:

*We are also a German company, but in Germany we use more than we do in Austria because we don’t have the BVS update yet. That’s why there are always exceptions, except in Austria, when using certain tools of SAP, for example. And in Germany it’s easier than in Austria. […] in the Unites States of America we have a full version of SAP. In Germany it’s less and here it’s a little less again and it will be the same for other companies. [E1]*

This quote indicates that the expert group is aware that legislation on AI regulation currently depends on national priorities and legal frameworks, which leads to varying degrees of restrictions on AI use. At the same time, there is also the fear of the expert group that despite the existing legal situation, this will not be fully considered when using AI. This is because organizations buy tools that are available on the market with a full version but may not be used in full in their country of location due to legal regulations. As the verification of legally compliant use, such as data protection, is often outsourced by organizations to external bodies, it is assumed that this is not carried out accordingly. Based on past experience with other HRM tools, the expert group expressed concerns that legal aspects were deliberately not considered or not tested within the organization when introducing HRM-specific AI systems until legal consequences arose. It is assumed that HRM tools such as AI are not used within the organization in a legally compliant way. This fear is based on past experiences that the expert group has had with reviewing the legally compliant use of various HRM tools within their organization:

*But since the new management and after the lawyers all quit, everyone assumes that we no longer need data protection. Because they don’t want to hear that, it [checking legal compliance] is outsourced and since then we have been doing what we want until we get a consequence. […] No, we have a work council agreement, it’s not like that. I always say, it says this and you have to do this and this and this. But I can’t always monitor it. [E10]*

This quote indicates that other HRM tools have not been sufficiently audited for compliance with the law, which is why this is also expected concerning using AI. Even if the assessment of the legally compliant use of AI in HRM is carried out within the organization, it is feared that this will not be done accordingly. Because even if they are audited for compliance, there are doubts as to whether this is being done by employees with the relevant expertise. The novelty and peculiarities of AI systems raise many complex and new legal issues, which is why specialized expertise and a sound understanding of specific laws and ethical guidelines are considered necessary by the expert group. It was assumed that organizations do not check whether the person responsible have the necessary skills or expertise:

*The reason will be the human who trains the AI. I got a job offer on LinkedIn for lawyers remotely in Graz. Which company offers lawyers a remote position in Graz? I looked through the English text once and was intrigued as to what I was supposed to do. I am a compliance officer for a company. Basically, they want nothing more than to give me data and to say from a legal perspective for Austria whether it is legally correct or not. I train artificial intelligence and I am a freelancer and get paid for it. […] I’m sure that if you have a few students who have just finished and want a bit of money, they’ll take it. But nobody knows whether their decisions are legally correct. [E10]*

This quote indicates the fear of the expert group that legal violations of the use of AI are partly due to a lack of legal expertise within the organization. There is also the fear that the use of AI is restricted by restrictive requirements imposed by the organization itself. This is because the expert group reported on several experiences in which the use of HRM tools was generally permitted in HRM from a legal perspective, but individual functions were prohibited within the organization in the form of a company agreement. Based on past experience, it is assumed that these incidents could be repeated concerning AI. Furthermore, the expert group fear that only limited benefits can be gained from AI because not all possible functions are made available within the organization.

Based on these concerns, the expert group named lawful AI as the second most important technical requirement for adopting AI. This requirement refers to compliance with a (higher-level) legal regulation for the use of AI systems before and during their use as well as technical implementation of the Labour Constitution Act and the works agreement in the AI system. It indicates both the need for legal protection and concerns that the special features of AI systems do not currently fulfill the legal standards.

*There is a work council agreement, without a work council agreement there is no AI. […] And the rest has to be clarified in the work council agreement. That would be a clear definition of the data sources. I would not say any external ones, but a clear definition of where the data comes from. Which data sources are used for the analysis? A clear definition of the use case thus where specifically is AI being used? Decisions support? Which elements of which decision-making basis does the career provide? If I say we have a basis for decision-making, which is a job setting and then we say a basis for decision-making is precisely that and the analysis […]. [E3]*

We identified promising measures in the literature that fulfill the requirement for considering legal regulations at three different levels: *Low level* includes trust marks and AI certification labelling (e.g., CE labelling) which indicate compliance with existing (AI) laws and regulations (Scharowski et al., 2023; Lu et al., 2024). *Medium level* also includes regular AI audits and conformity assessments to verify that the AI system complies with AI standards and regulations. *High level* provides the possibility of defining agreements beyond the legal requirements on topics such as clear definition of data sources, data storage and management, data erasure, and proportion of the basis for decision-making.

There was a clear consensus within the expert group that the possibility of defining agreements that go beyond the legal requirements (*high level*) was considered the most suitable measure to ensure lawful AI. It was justified by the fact that laws can change over time, which is why it is important to be able to adapt agreements. Despite the clear consensus, *low level* and *medium level* were also discussed as suitable measures. Here, the demand for the regular performance of AI audits and conformity assessments (*medium level*) to ensure lawful AI was explained as follows:

*When implementing legal regulations, I assumed level 2 concerning the AI Act, which was decided by the EU, which already contains many precautions and already includes what level 3 says as a possibility. […] it is already included, so level 2 is enough. [E10]*

This statement indicates that audits can provide assurance that the AI system fulfills all requirements for the legally compliant use of AI. Furthermore, it indicates that it is assumed that AI regulations have already taken sufficient precautions in this regard.

#### 4.2.3 TR3: data security

In the focus group workshops, the handling and processing of HRM-specific data by AI systems played a central role from both a technical and legal perspective, which was also linked to concerns. This is because sensitive and personal information is processed within the various HRM areas, and the expert group fears that AI will not process this in a legally compliant or secure manner. There are concerns that AI systems are based on cloud infrastructures and therefore (unintentionally) access external data to perform HRM tasks. This fear is exacerbated by the expert group’s assumption that the data sources of AI systems cannot be traced, meaning that this concern is linked to the black-box nature of AI. This fear is particularly present in the context of employee appraisals and evaluations:

*No external data should be used. In general. Once I have the person customer, I can continue searching. I can search for the data not only in the company, but also on the entire internet. And that was critical. [E1]*

This quote highlights the expert group’s preference for AI systems to process locally available or strictly controlled data, as this can protect the security and privacy of HRM stakeholders. Access to external data is perceived as risky, as these conditions encourage the unauthorized processing of sensitive and personal information. Concerns about using AI in HRM relate to processing, storing, and deleting of HRM-specific data. There are fears that the use of AI may neglect to comply with legal data protection regulations, for example by not deleting the data at all or not deleting it within the legally prescribed timeframe.

*[…] It should have an automated data deletion that is irretrievable. You are allowed to keep applicant data for a maximum of six months and then it must be deleted. And it has to be done in such a way that it is done carefully. [E10]*

This quote indicates that the expert group is aware of the legal provisions on data security, but it is unclear to what extent they (can) be fulfilled by AI. This is one of the reasons why the corresponding maintenance of AI systems and the implementation of updates is a high priority within the expert group.

Based on the above-mentioned concerns about data security, the expert group named data security together with lawful AI as the second most important technical requirement for adopting AI in HRM. Data security describes that data is protected by avoiding (unwanted) external data exchange, providing data storage and management information, and ensuring legally compliant data deletion. This requirement also includes ensuring that AI systems are maintained and updated. The demand for data security indicates a growing awareness of the need to protect sensitive and personal information processed by the AI system and concerns about compliance with legal data protection requirements.

To identify the state-of-the-art approaches already available in the literature to fulfill these requirements, we examined them for promising measures. We identified three promising measures that fulfill the requirement for data security at three different levels. *Low level* proposes that data security is integrated into the existing AI landscape by the AI provider. *Medium level* involves the performance of an internal audit, meaning that the AI system is additionally certified by an internal auditor (organization using AI or AI provider) (Liu et al., 2022; Raji et al., 2020). *High level* includes the separate external certification of the AI component. As there is independent oversight from experts with expertise in conducting audits who have no conflict of interest with the audited organization, a trustworthy audit infrastructure is provided that increases the trust in AI (Lu et al., 2024).

We discussed the measures identified in the literature with the expert group to determine the extent to which they were considered suitable for meeting their data security requirements. There is a consensus that a separate external certification of the AI components (*high level*) is considered the most suitable. Independent oversight was considered to be required, as it is feared that there may be attempts to conceal and manipulate the results of audits that are performed in-house:

*As far as data security is concerned, […] I don’t think it’s possible without external certification, because there’s simply too much in-house cheating. [E10]*

Even though there is a clear consensus regarding *high level*, *medium level* (certification by an internal auditor) was also discussed as fulfilling the requirement. *Low level* including that data security is integrated into the existing AI landscape by the AI provider was not perceived by any expert group member as a suitable measure.

## 5 Discussion

This paper tackles one of the core issues in the regulatory efforts of AI in HRM: *How can the adoption of AI in HRM be designed to ensure a balance between employee and organizational interests?* We developed a stakeholder-oriented catalog for adopting AI in HRM listing both requirements and promising measures to fulfill them from the perspective of workers’ representatives. We find that workers’ representatives perceive the use of AI in HRM as critical, with concerns varying for the different ELC stages and being expressed to varying degrees. Concerns are most pronounced in *personnel selection* and include perceived lack of transparency, responsibility, objectivity, and neglect of human oversight. Based on the varying concerns of workers’ representatives when using AI in the individual ELC stages, we identified six organizational and technical requirements for adopting AI in HRM, and perceived suitable measures to fulfill them. Our developed requirement-measure-catalog is displayed in Figure 2.

### 5.1 Theoretical contribution

We advances the AI in HRM research by investigating the requirements from an under-researched key HRM stakeholder group. The EU’s AIHLEG has—using a bottom-up approach and thus in consultation with various stakeholders—developed seven requirements for the responsible use of AI in high-risk areas such as HRM. Despite this stakeholder-oriented approach of the AI-HLEG, stakeholders have different interpretations of these ethical principles for responsible AI, which also vary depending on the area of application (Figueras et al., 2022). This plethora of interpretations is a challenge, made even more critical in HRM, where the perspectives of key HRM stakeholders such as workers’ representatives have been simply unknown. In our study, we identify and prioritize the requirements and suitable measures for AI in HRM, from the perspective of workers’ representatives. Based on our findings, we can describe requirements for responsible AI from workers’ representatives’ perspective. Three requirements can be categorized as AI compliance: (1) control (OR1), (2) lawful AI (TR2), and data security (TR3) (see Figure 2). Those three requirements identified in the focus group workshops primarily result from concerns that workers’ representatives have regarding AI’s technological readiness to perform certain tasks or legal non-compliance of AI system components. This is also demonstrated that these requirements/concerns arise in various fields of application. For example, in medicine, the risk of biases in the data leading to inaccurate medical results (OR1—control) is discussed (Grzybowski et al., 2024), while in the financial sector it is criticized that the data used by AI is not processed following the law (TR3—data security) (Deshpande, 2024) and is therefore perceived as a requirement in different disciplines. The AI compliance requirements identified in the focus groups—control (OR1), lawful AI (TR2), and data security (TR3)—mirror requirements of the AI Act: (1) risk management system, (2) data and data governance, (3) technical documentation, (4) record-keeping, and (5) accuracy, robustness, and cybersecurity. Like our identified requirements, the AI Act requirements mentioned can also be assigned to the AI Compliance category. This is reflected in the literature on adopting AI in HRM: these are predominantly technical topics mainly discussed in the context of the IT infrastructure (e.g., Wilson et al., 2021). Furthermore, a study in which participants were asked about the perceived importance of the individual ethical principles of the AI Act for their work in developing AI systems for the public sector revealed that those such as technical robustness are taken for granted (Figueras et al., 2022). The technical consideration in the literature and the assessment of certain requirements of the AI Act as standard practice indicate that these reflect fundamental principles that play a central role in various disciplines. Our study showed that workers’ representatives consider the identified requirements to be fulfilled if the verification of compliance is outsourced to an external body and confirmed by certifications. It can be assumed that this assessment is due to the cross-disciplinary nature of the identified requirements.

The second category of requirements identified for adopting AI in HRM relates to the interaction between humans and AI. We identify three requirements that can be assigned to this category: (1) transparency and explainability (TR1), (2) human oversight (OR2), and (3) responsibility (OR3). The category interaction between humans and AI is characterized by its assigned requirements being specifically oriented toward the use of AI in HRM (HRM-specific).

Our study revealed that the requirement transparency and explainability was named as the most important technical requirement by workers’ representatives. This requirement reflects the basic idea of the AI Act requirement transparency and provision of information to users, which we have concretized for specific use cases in HRM. This AI Act requirement includes a transparent design and development of AI systems, including the provision of complete information about the AI system and the option that the AI-based outcome can be interpreted by users (Article 13 of the AI Act) (European Parliament, 2024). In the HRM context, workers’ representatives fear that AI will recognize bias in the data, generating discriminatory personnel selection decisions that cannot be understood due to its black-box nature. If AI-based personnel selection decisions cannot be understood by workers’ representatives, this limits their ability to veto HRM decisions and making it unclear who is responsible for incorrect and sometimes unlawful AI-based outcomes. Workers’ representatives classify the requirement as fulfilled if the decision variables for AI-based HRM decisions are communicated transparently (medium level). The requirement for transparency and explainability is already discussed in the literature, but the important perspective of workers’ representatives is so far missing. In the literature, explainability refers to the understanding how an AI model makes decisions, while transparency refers to the disclosure of information about the life cycle of an AI system (Li et al., 2023). The requirements for explainability and transparency vary depending on the key HRM stakeholder groups. Basically, depending on who it is to be explained to, what is to be explained, in what type of situation it is to be explained, and who is explaining, the requirement must be addressed in different ways (Balasubramaniam et al., 2023). While AI operators use explainability to understand the AI system to prevent defects, users such as applicants want to be able to understand AI-based rejections (Li et al., 2023).

Human oversight (QR2) was named the second most important organizational requirement for the adoption of AI by the workers’ representatives. Our study revealed that the workers’ representatives’ understanding of human oversight is mainly in line with that of the AI Act (Article 14 of the AI Act) (European Parliament, 2024) but still focuses on HRM-specific aspects. The need for human oversight is perceived by workers’ representatives primarily in the ELC stages personnel selection, retention and HR analyses, and development and training is justified by the assumption that HRM employees rely too much on AI-based personnel selection decisions and that organizations do not counteract this sufficiently due to effort and cost factors. It is also assumed that AI cannot recognize human components when selecting candidates or building work teams, which means that promising candidates are unintentionally rejected or incompatible work teams are formed. We find the assumption that HRM decisions are very sensitive and therefore require human expertise and control. Human oversight is discussed in the literature on adopting AI depending on the stakeholder group (Laurim et al., 2021). For example, HR professionals are seeking to maintain a sense of control over the AI system (Malin et al., 2023). In the literature, the degree of control or stakeholder involvement in AI systems is considered an essential element for the responsible use of AI (Bujold et al., 2023). Its relevance is reflected in the legal situation, as from a legal perspective, human oversight has been prescribed in the form of several regulations such as §22 of the General Data Protection Regulation (GDPR), in that the final decision must still be made by a human. However, studies indicate that overreliance on AI systems is very common (e.g., Malin et al., 2024). An indicator that the balance between complementing the human factor without overshadowing it remains a challenge despite legal efforts (Rane, 2023). Many people are afraid that they will lose control of AI and that humans will be replaced by it (Lukaszewski and Stone, 2024).

Responsibilities (OR3) was mentioned as the third most important organizational requirement not directly matching an AI Act requirement. From a legal perspective, upcoming regulations (AI Act) or industry-specific laws are currently being worked on to regulate the responsibilities for developing, using, and monitoring AI. The unauthorized access to (employee) data is seen as a key challenge to the use of AI in HRM (Du, 2024) affecting the accountability (Du, 2024). Stakeholders along the AI pipeline have different perspectives on accountability, explaining why it is addressed differently in research and practice (Srinivasan and González, 2022). For example, regulatory authorities must be able to assess the AI system for legal compliance, decision-makers must be able to justify AI-based decisions, and consumers must be able to correct adverse decisions (Srinivasan and González, 2022). Although various key HRM stakeholders (e.g., employees) call for defined responsibilities, from a legal perspective it is still unclear who is responsible for AI-based decisions (Hunkenschroer and Luetge, 2022). As AI differs from conventional HRM tools, workers’ representatives assume that HRM employees are not adequately trained to maintain or update AI systems. Therefore, the assignment of competent (non-HRM) persons is required to perform these tasks.

### 5.2 Practical implications

Our findings have direct relevance for the work of the European Commission, regulatory authorities, and standardization committees. Currently, the AI Act regulations are being specified considering feedback from stakeholders whose interests have so far been analyzed to varying degrees in research on AI in HRM. The uneven distribution of attention in research poses a problem as important stakeholders’ perspectives may be underrepresented. Our study provides a comprehensive view of the requirements of workers’ representatives as a previously under-researched HRM stakeholder group, revealing how and why they perceive using AI in certain HRM phases critically. Our findings help the relevant parties to better understand the needs of this specific stakeholder group and to address them from a practical perspective.

The presented requirement-measure-catalog can serve as a platform for regulators and organizations to develop technical standards and solutions, and the stakeholder-driven development of HRM-specific AI systems. The use of AI is often viewed critically by key HRM stakeholders, which can negatively impact its adoption in organizations (e.g., Malin et al., 2023): When expectations and interests of stakeholders are not sufficiently considered, aversion to AI can be the result (Jussupow et al., 2020). Research increasingly suggests that stakeholder interests should already be considered in developing solutions and AI systems (Mahmud et al., 2022). Our study identifies several factors that encourage critical perception, and requirements and measures to fulfill them. Knowledge of these factors enables targeted action, as regulators and organizations can now better understand requirements of HRM-specific AI systems from the perspective of workers’ representatives.

Our results can also serve as orientation for organizations to ensure that the interests of employees are met. Our requirements can be seen as categories that need to be specified in the context of the organization and the concrete AI system. Such resulting criteria are relevant for both AI acceptance and legal compliance. From a legal perspective, workers’ representatives have rights of co-determination and participation in adopting AI in HRM. For example, the introduction and use of technical equipment designed to monitor the behaviour or performance of employees (§ 87 (1) No. 6 BetrVG), it has a right to information and consultation when planning the use of AI (§ 90 BetrVG) and it is regulated that the introduction of AI may be involved in selection guidelines for personnel decisions, such as recruitment or promotion (§ 95a (2) BetrVG). Even if the scope of legal action for the use of AI still needs to be clarified, the aforementioned provision makes it clear that the requirements of this stakeholder group must be considered.

### 5.3 Limitations

This study has six main limitations. First, not all experts had an active work council function at the time of the study. However, they attended trade union schools, where they were preparing to take up this role soon. Thus, we are confident they are a suitable sample that reflects the target group under investigation.

Second, the findings gathered using focus group workshops can be biased by group dynamics, the dominance of individual participants and social desirability, leading to stereotypical and insincere responses (Acocella, 2012; Nyumba et al., 2018). To minimize these limitations, the focus group workshops were moderated by the authors, who actively strived to ensure a balanced discussion. All participants were encouraged to interact by asking specific questions, and interactive methods such as brainstorming with index cards and anonymous voting were used to encourage the participation of all participants and reduce social desirability bias.

Third, workers’ representatives might be biased towards adopting AI in HRM, seeing AI primarly as a threat to jobs, co-determination and data protection, while giving less consideration to potential opportunities such as efficiency benefits or positive impacts on employees (Bozkurt and Gursoy, 2023). To counteract these biases, we conducted a training workshop to inform the workers’ representatives about AI functionalities, possible applications and impacts. Furthermore, when selecting the participating workers’ representatives, we ensured a broad coverage of different organizational areas. This ensured different perspectives of this stakeholder group, for example from lawyers, technology experts and other types of employees, to be included in the discussion and promoted a balanced viewpoint.

Fourth, our findings can only be generalized to a limited extent to other regulatory environments or specific regions to the strict regulatory setting and the associated high requirements in the European Union. These specific requirements may not be captured in the same way in less strictly regulated contexts. This also applies to our sample, which is located in this specific regulatory environment and therefore reflects the special characteristics and requirements of this environment.

Fifth, the identified requirements of the expert group are on a conceptual level and not directly implementable. To minimize this limitation, we have identified promising measures for fulfilling the requirements in the literature. The measures proposed in the literature are well-studied and proven solutions and show the extent to which the requirements can be met in practice. We also discussed the requirements with a provider of AI HRM solutions. This person rated the requirements as suitable to instantiate HRM AI solutions in practice and saw no further requirements.

Sixth, the workers’ representatives were not experts in the AI field, as they had different backgrounds. To ensure a (common) understanding of the basics and functions of AI, we conducted a training workshop with them and provided information material tailored to the target group.

## 6 Conclusion and directions for future research

The stakeholder-oriented approach of the AI Act is a first step toward applying HRM-specific AI in a regulated and accepted environment. Our study sheds nuanced light on a previously under-researched key HRM stakeholder group’s perception of adopting AI in HRM. Our results indicate that workers’ representatives perceive the adoption of AI in HRM critically, especially for personnel selection. We identified organizational and technical requirements for adopting AI in HRM and promising measures to meet them. We are confident that our findings will support global regulatory efforts and stakeholder-driven development of AI systems.

Given this background, our findings offer promising approaches for future research. It would be interesting to investigate how the requirements and measures for adopting AI in HRM identified in this study can be implemented in organizations. More specifically, to ensure successful implementation, light should be shed on how these findings could be integrated into existing organizational processes. Future research could develop concrete recommendations on how organizations can integrate the study findings into their AI governance and HRM practices to maintain both ethical standards and employee acceptance.

Furthermore, as our findings highlight challenges of adopting AI in HRM, they provide a promising basis for future research to investigate how to mitigate the effects of errors in AI systems in HRM and better understand the long-term impact on decisions. For example, Bélisle-Pipon et al. (2023) emphasize that, given the black-box nature of AI, the transparency of AI decisions needs to be improved. In addition, research should be conducted into how the interests of employees can be protected despite the use of AI, as principles alone do not guarantee ethical AI (Mittelstadt, 2019). Future studies should develop ethical and regulatory frameworks to ensure fair and transparent AI decisions in HRM.

## Data Availability

The original contributions presented in the study are included in the article/supplementary material, further inquiries can be directed to the corresponding author.
